# Cellular messengers involved in the inhibition of the Arabidopsis primary root growth by bacterial quorum-sensing signal *N*-decanoyl-L-homoserine lactone

**DOI:** 10.1186/s12870-022-03865-6

**Published:** 2022-10-14

**Authors:** Xiang-yu Cao, Qian Zhao, Ya-na Sun, Ming-Xiang Yu, Fang Liu, Zhe Zhang, Zhen-hua Jia, Shui-shan Song

**Affiliations:** 1 Biology Institute, Hebei Academy of Sciences, 46th, South Street of Friendship, 050051 Shijiazhuang, Hebei China; 2 Hebei Engineering and Technology Center of Microbiological Control on Main Crop Disease, 46th South Street of Friendship, Shijiazhuang, China; 3 College of Life Science, Hebei University, 180th East Road of Wusi, Baoding, China; 4 Hebei Collaboration Innovation Center for Cell Signaling Environmental Adaptation, 20 East NanErhuan Road, Shijiazhuang, China

## Abstract

**Background:**

*N*-acyl-homoserine lactones (AHLs) are used as quorum-sensing signals by Gram-negative bacteria, but they can also affect plant growth and disease resistance. *N*-decanoyl-L-homoserine lactone (C10-HSL) is an AHL that has been shown to inhibit primary root growth in *Arabidopsis*, but the mechanisms underlying its effects on root architecture are unclear. Here, we investigated the signaling components involved in C10-HSL-mediated inhibition of primary root growth in *Arabidopsis*, and their interplay, using pharmacological, physiological, and genetic approaches.

**Results:**

Treatment with C10-HSL triggered a transient and immediate increase in the concentrations of cytosolic free Ca^2+^ and reactive oxygen species (ROS), increased the activity of mitogen-activated protein kinase 6 (MPK6), and induced nitric oxide (NO) production in *Arabidopsis* roots. Inhibitors of Ca^2+^ channels significantly alleviated the inhibitory effect of C10-HSL on primary root growth and reduced the amounts of ROS and NO generated in response to C10-HSL. Inhibition or scavenging of ROS and NO neutralized the inhibitory effect of C10-HSL on primary root growth. In terms of primary root growth, the respiratory burst oxidase homolog mutants and a NO synthase mutant were less sensitive to C10-HSL than wild type. Activation of MPKs, especially MPK6, was required for C10-HSL to inhibit primary root growth. The *mpk6* mutant showed reduced sensitivity of primary root growth to C10-HSL, suggesting that MPK6 plays a key role in the inhibition of primary root growth by C10-HSL.

**Conclusion:**

Our results indicate that MPK6 acts downstream of ROS and upstream of NO in the response to C10-HSL. Our data also suggest that Ca^2+^, ROS, MPK6, and NO are all involved in the response to C10-HSL, and may participate in the cascade leading to C10-HSL-inhibited primary root growth in *Arabidopsis.*

**Supplementary Information:**

The online version contains supplementary material available at 10.1186/s12870-022-03865-6.

## Introduction

Many Gram-negative bacteria use *N*-acyl-homoserine lactones (AHLs) as signaling molecules for intercellular communication in a process known as quorum sensing (QS) [1–3]. To date, numerous AHL derivatives with acyl chains of different lengths (from 4 to 18 carbons) and substitutions of hydroxyl or oxo groups at the γ position of the carbon chain has been identified from more than 70 species of Gram-negative bacteria [2, 4]. AHL-mediated QS plays an essential role in many bacterial physiological processes including symbiosis, virulence, antibiotic production, extracellular polysaccharide production, resistance to oxidative stress, biofilm formation, and motility [5–7].

Plants can also sense and respond to bacterial QS signals [8–12]. For example, Mathesius et al. (2003) found that more than 150 proteins in *Medicago truncatula* changed in abundance after application of 3-oxo-*N*-(tetrahydro-2-oxo-3-furaryl)-hexadecenamide (3OC16-HSL) isolated from cultured *Sinorhizobium meliloti* [13]. Schuhegger et al. (2006) demonstrated that *N*-hexanoyl-homoserine lactone (C6-HSL) induces systemic accumulation of salicylic acid- and ethylene-dependent defense gene transcripts [14]. In *Arabidopsis, N*-3-oxo-tetradecanoyl-homoserine-lactone (3OC14-HSL) conferred resistance to biotrophic and hemibiotrophic pathogens *via* an oxylipin/SA-dependent pathway [9]. Whereas long-chain AHLs (side chains with 12–14 carbons) induce defense responses, short-chain AHLs (side chains with fewer than 8 carbons) promote elongation of the plant primary root. The G-protein-coupled receptor (GCR) and the transcription factor AtMYB44 were involved in the regulation of primary root growth by 3-oxo-octanoyl-homoserine lactone (3OC8-HSL) [15, 16].

Medium-chain AHLs, i.e., those with 10 carbon atoms in the carbon chain with or without substitutions at the γ position, have multiple functions in root morphogenesis, plant senescence, and defense responses [11, 17–20]. Hu et al. (2018) reported that *N*-decanoyl-homoserine lactone (C10-HSL) activates plant systemic resistance against *Botrytis cinerea* in tomato, and that C10-HSL-induced resistance is largely dependent on the jasmonic acid (JA) signaling pathway [19]. Ortiz-Castro et al. (2008) compared the effects of seven different AHLs on root architecture in *Arabidopsis* and found that C10-HSL had the strongest effect to inhibit primary root growth and promote lateral root formation [18]. Bai et al. (2012) found that an analog of C10-HSL, *N*-3-oxo-decanoyl-homoserine lactone (3OC10-HSL), mediated auxin-dependent adventitious root formation *via* H_2_O_2_- and nitric oxide (NO)-dependent cGMP signals in mung bean (*Vigna radiata*) seedlings [11]. However, the signaling pathway through which C10-HSL modulates plant primary root growth remains unclear.

Reactive oxygen species (ROS) and NO are important signals that participate in various physiological processes [14, 18, 21–26]. Zhang et al. (2017) found that ROS and NO were involved the inhibition of primary root growth by exogenous H_2_S in *Arabidopsis* [27]. Treatments with C6-HSL, *N*-octanoyl-homoserine lactone (C8-HSL), C10-HSL, and *N*-decanoyl-homoserine-lactone (C12-HSL) led to NO accumulation in the calyptra and elongation zone of barley roots and altered root morphology [28]. Recently, we reported that pretreatment with 3OC8-HSL and subsequent pathogen invasion triggered an augmented ROS burst in *Arabidopsis* [29].

Mitogen-activated protein kinase (MAPK) cascades are highly conserved signal transduction pathways that participate in the regulation of growth and development and in responses to environmental stress in plants [30–34]. AtMPK3/6 regulate plant growth, development, and stress tolerance by interacting with Ca^2+^ and ROS pathways [31, 35–37]. Schikora et al. (2011) found that 3OC14-HSL pretreatment activated AtMPK3/6 upon challenge by flg22 in *Arabidopsis* [9].Calcium ions (Ca^2+^) are a ubiquitous intracellular second messenger that participate in many signal transduction pathways in plants [38–41]. In our previous study, we found that C4-HSL induced a transient and immediate increase in the cytosolic free Ca^2+^ concentration ([Ca^2+^]_cyt_) in *Arabidopsis* [10]. Overall, data from the literature suggest that Ca^2+^, ROS, NO, and MAPK participate in the plant’s response to bacterial AHLs. However, the details of their involvement and the interplay among them in the regulation of primary root growth by C10-HSL is unknown. In this study, we investigated the involvement of intracellular Ca^2+^, ROS, NO, and MPKs in C10-HSL-inhibited primary root growth in *Arabidopsis.* Our data indicate that C10-HSL inhibits primary root growth *via* a cascade involving Ca^2+^, ROS, MAPK, and NO in *Arabidopsis.*

## Results

### C10-HSL influences ***Arabidopsis*** root system architecture

**Fig. 1:**
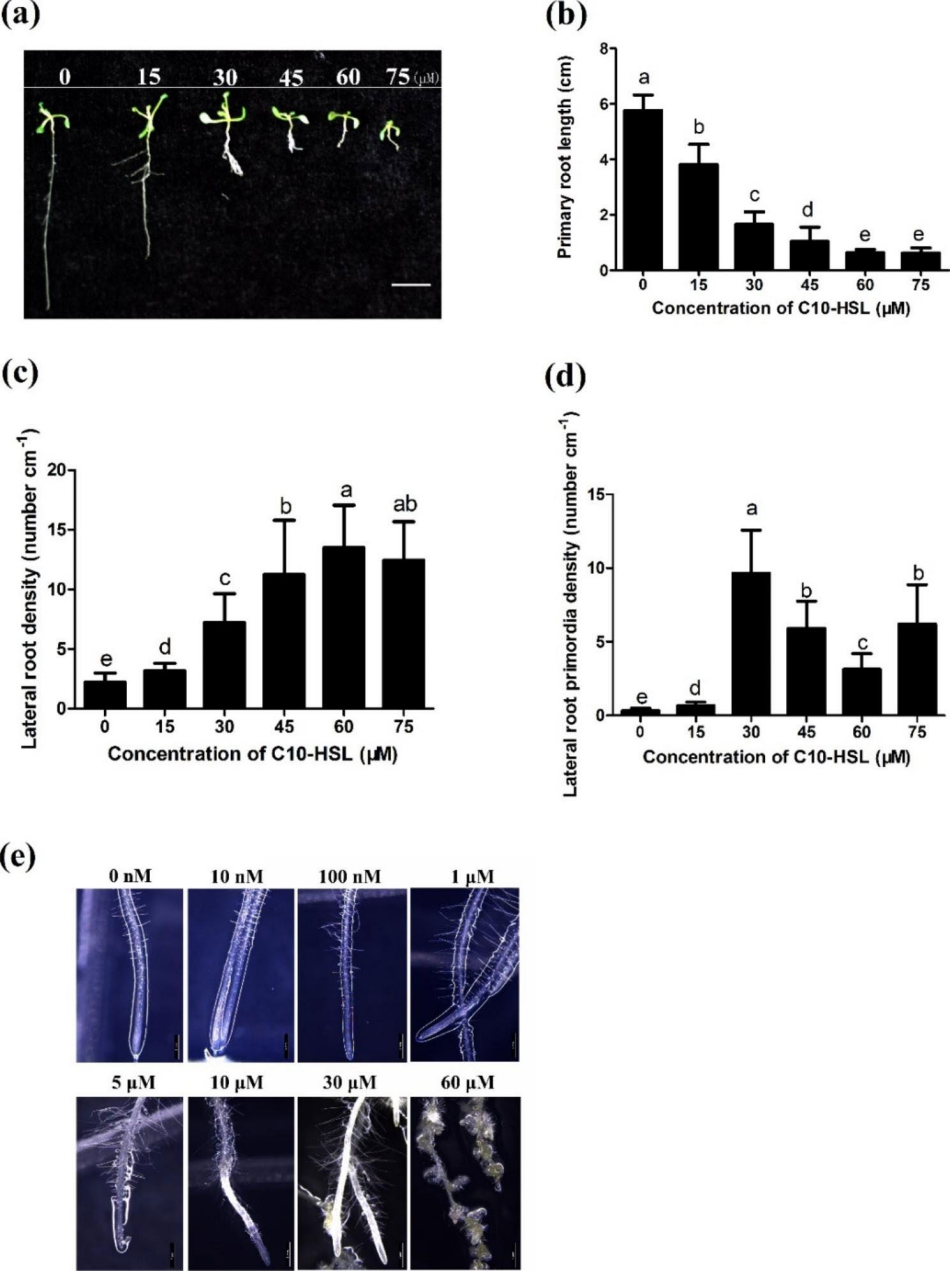
* N*-decanoyl-homoserine lactone (C10-HSL) influences root system architecture in Arabidopsis. **(a)** Root architecture of wild-type (Col-0) Arabidopsis treated with different C10-HSL concentrations (0–75 µM), bar = 1 cm. **(b)** The effects of different C10-HSL concentration on primary root length, n = 40. **(c)** The effects of different C10-HSL concentration (0–75 µM) on lateral root density, n = 40. **(d)** The effects of different C10-HSL concentration (0–75 µM) on lateral root primordia density, n = 40. For the picture a-d, 3-d-old *Arabidopsis thaliana* seedlings were transferred to half-strength MS medium supplemented with the indicated concentrations of C10-HSL and grown for 5 d. **(e)** Representative photographs of root hairs formed at the primary root tip region of 3-day-old Arabidopsis seedlings grown in the presence of the indicated concentration of C10-HSL for 5 d, bar = 1 mm. All the error bars represent +/- SD. (Different letters indicate significantly different values, P < 0.05 by Tukey’s test)

Previously, Ortiz-Castro et al. (2008) reported that C10-HSL is one of the most active AHLs tested to date, in terms of modification of *Arabidopsis* root system architecture [18]. In this study, we first confirmed the effects of C10-HSL on post-embryonic root development. We found that 10 nM to 1 µM C10-HSL treatment slightly caused a reduction in primary root growth and promoted lateral root formation (Fig. S1a, b). The primary root growth of plants treated with 15 to 75 µM C10-HSL was inhibited by 40–86% (Fig. 1a, b) and treatment 30 µM C10-HSL caused a 74% reduction in primary root length. Treatment with C10-HSL also resulted in significant increases in lateral root density and the density of lateral root primordia (Fig. 1c–e and Fig. S1a). These data confirmed that C10-HSL can inhibit primary root growth and stimulate lateral root formation.

### NO is involved in the inhibition of primary root growth by C10-HSL

**Fig. 2:**
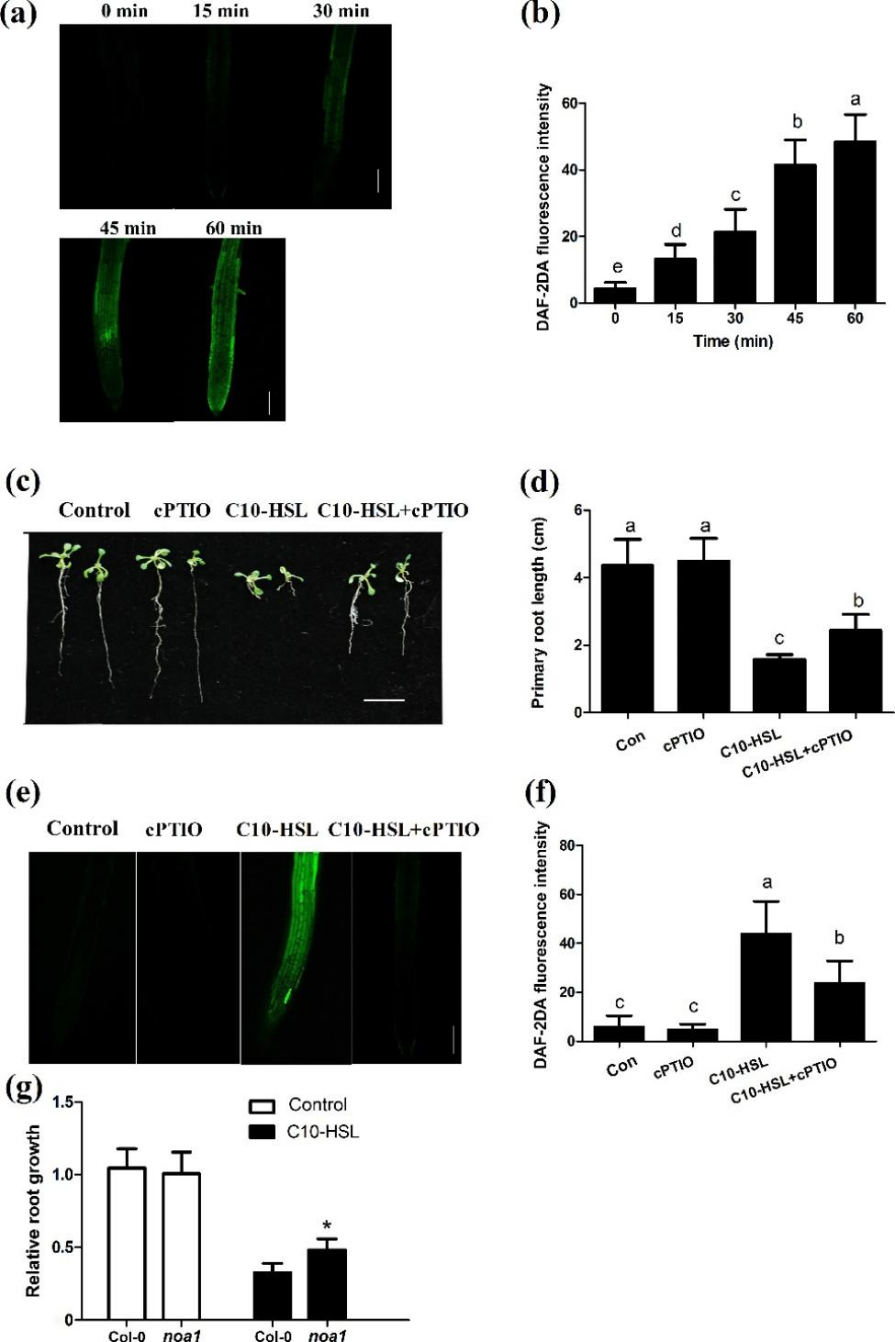
NO is involved in the inhibition of primary root growth mediated by C10-HSL. **(a, b)** Detection of NO production in roots of Col-0 seedlings treated with C10-HSL (0–75 µM) for 0–60 min using the NO-specific fluorescent probe DAF-2DA (a) and quantification of NO-specific fluorescence intensities (b), bar = 50 μm, n = 30. **(c)**, Root architecture of wild-type Arabidopsis exposed to 30 µM C10-HSL with or without 100 µM cPTIO, bar = 1 cm. **(d)** Primary root length of wild-type seedlings exposed 30 µM C10-HSL with or without 100 µM cPTIO, n = 40. **(e, f)** Detection of NO production in roots of Col-0 seedlings treated as described in (c) using the NO-specific fluorescent probe DAF-2DA (e) and quantification of NO-specific fluorescence intensities (f). **(g)**, Relative primary root growth of wild-type and *noa1* exposed to 30 µM C10-HSL, n = 40. For the picture c, d and g, 3-d-old *Arabidopsis thaliana* seedlings were transferred to half-strength MS medium supplemented with the indicated concentrations of C10-HSL or other chemicals and grown for 5 d. Control or Con refers to solvent control. All the error bars represent +/- SD. (Different letters indicate significantly different values, *P* < 0.05 by Tukey’s test; *P < 0.05, **P < 0.001, Student’s t test)

Nitric oxide is an important gaseous signal that regulates root development [23, 42, 43]. To investigate the role of NO in C10-HSL-induced inhibition of primary root growth, we first measured NO production after C10-HSL treatment in *Arabidopsis* seedlings. Using the NO specific fluorescent probe diaminofluorescein diacetate (DAF-2DA), we found that C10-HSL elicited NO production in the roots within 15 min of treatment, and this effect lasted for 1 h or even longer (Fig. 2a, b). Supplementation with 100 µM 2-4-carboxyphenyl-4,4,5,5-tetramethylimidazoline-1-oxyl-3-oxide (cPTIO), an NO-specific scavenger, markedly alleviated the C10-HSL-induced inhibition of primary root growth (Fig. 2c, d). Consistent with the phenotypic results, NO production induced by C10-HSL treatment was significantly suppressed by cPTIO (Fig. 2e, f). We further analyzed the transcript levels of *NOA1*, encoding cGTPase [44], and *NIA1* and *NIA2*, encoding nitrate reductase, after treatment with C10-HSL in wild-type *Arabidopsis*. After C10-HSL treatment, the transcript level of *NOA1* increased within 1 h, peaked at 6 h after treatment, and then gradually declined (Fig. S2f). Similarly, the transcript levels of *NIA1* and *NIA2* increased within 1 h of treatment with C10-HSL in *Arabidopsis* roots (Fig. S2a, b). Seedlings of the *noa1* mutant were less sensitive to C10-HSL treatment with respect to primary root growth (Fig. 2 g). Likewise, *nia1* and *nia2* single mutants and *nia1nia2* double mutants were less sensitive to inhibition of primary root growth by C10-HSL (Fig. S3e). Collectively, these data suggest that NO is involved in the C10-HSL-mediated inhibition of primary root growth.

### ROS are essential in C10-HSL-induced inhibition of primary root growth

**Fig. 3:**
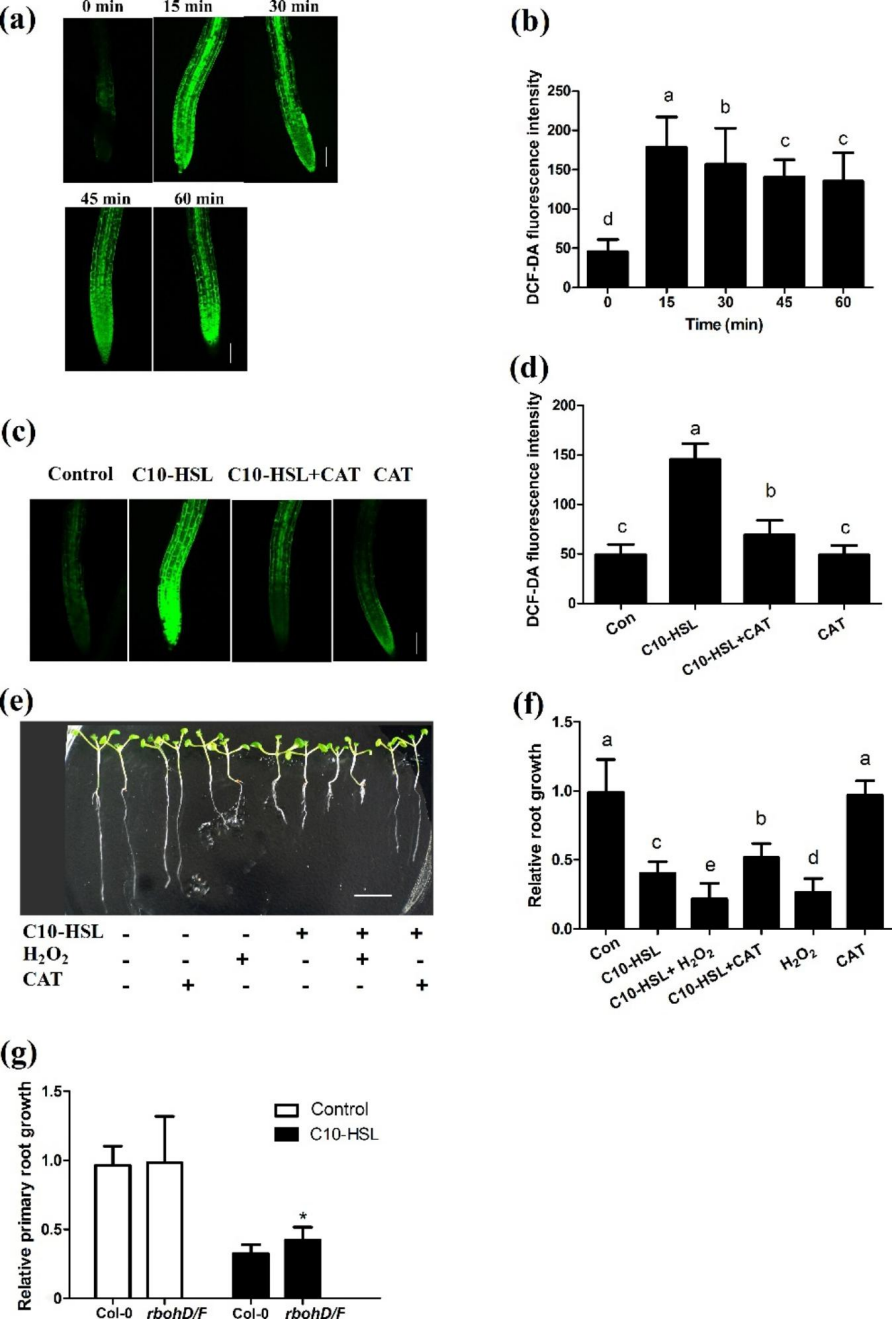
ROS is involved in C10-HSL-mediated inhibition of primary root growth. **(a, b)** Detection of ROS level in the roots of wild-type seedlings exposed to 30 µM C10-HSL for 0–60 min using the ROS-specific florescent probe DCF-DA (a), and quantification of ROS-specific fluorescence intensities (b) bar = 50 μm. **(c, d)** Detection of ROS level in the roots of wild-type seedlings exposed to 30 µM C10-HSL with or without 500 µM CAT for 15 min using the ROS-specific florescent probe DCF-DA (c), and quantification of ROS-specific fluorescence intensities (d), n = 30, bar = 50 μm. **(e, f)** Relative primary root growth of wild-type seedlings exposed to 30 µM C10-HSL with or without 1.5 mM H_2_O_2_ and 500 µM CAT for 5 d, bar = 1 cm, n = 40. **(g)** Relative primary root growth of Col-0 and *noa1* seedlings exposed to 30 µM C10-HSL for 5 d, n = 40. Control or Con refers to solvent control. All the error bars represent +/- SD. (Different letters indicate significantly different values, *P* < 0.05 by Tukey’s test. *P < 0.05, **P < 0.001, Student’s t test)

In plants, ROS play an essential role in root growth and development [25, 45, 46]. To investigate the role of ROS in C10-HSL-mediated inhibition of primary root growth, we determined their levels in C10-HSL-treated seedlings using the ROS-specific fluorescent probe 2’,7’-dichlorofluorescein diacetate (DCF-DA). The ROS levels in wild-type seedlings treated with 30 µM C10-HSL increased and peaked at 15 min after treatment (Fig. 3a, b). One important component of ROS is H_2_O_2_, which can be scavenged by catalase (CAT). When CAT was added to the medium containing 30 µM C10-HSL, the ROS level was 30% lower than that in the control (solvent-treated) seedlings (Fig. 3c, d). Then, we examined primary root elongation in wild-type seedlings treated with 30 µM C10-HSL in the presence or absence of exogenous H_2_O_2_ and CAT. We found that the C10-HSL-induced inhibition of primary root growth was increased by exogenous H_2_O_2_, but alleviated by CAT (Fig. 3e, f). The effect of C10-HSL on primary root growth was similar to that of H_2_O_2_, because addition of H_2_O_2_ alone significantly inhibited primary root growth (Fig. 3e, f). To verify the role of ROS in C10-HSL-mediated primary root growth inhibition, we analyzed the *Arabidopsis* ROS-biosynthesis-related respiratory burst oxidase homolog (Rboh) NADPH oxidase double mutant *rbohD/F*. In terms of the inhibition of primary root growth, *rbohD/F* plants were less sensitive to C10-HSL treatment than were wild-type plants (Fig. 3 g). These data indicate that ROS plays an essential role in the modulation of root growth by C10-HSL.

### MAPKs participate in the inhibition of primary root growth by C10-HSL

**Fig. 4:**
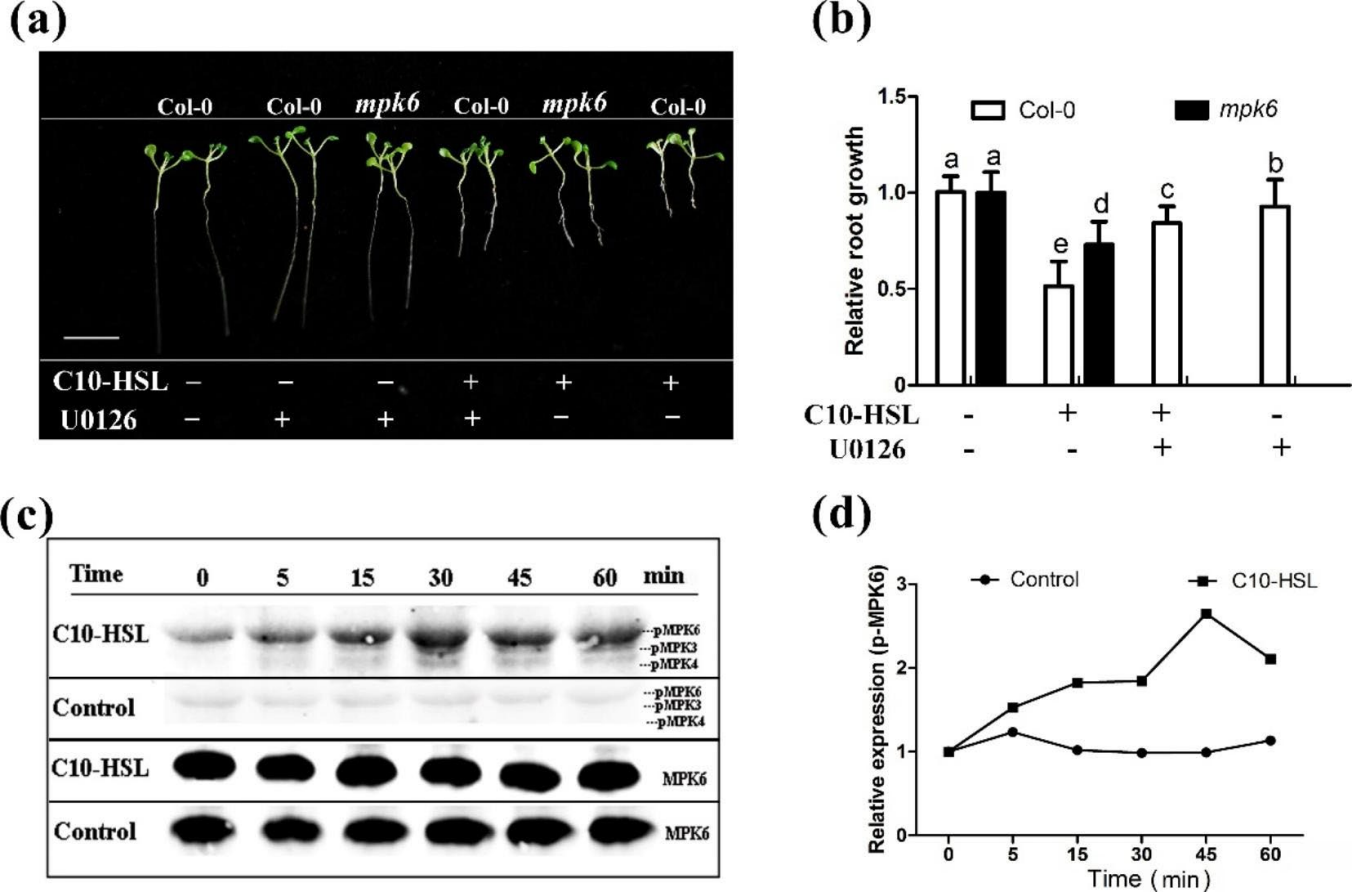
MAPKs participates in the inhibition of primary root growth by C10-HSL. **(a, b)** Relative primary root growth of Col-0 and *mpk6* seedlings exposed to 30 µM C10-HSL with(+) or without(-) 20 µM U0126 (MAPK inhibitor) for 5 d, n = 40, bar = 1 cm. **(c)** Phosphorylation status (pMPK6, pMPK3 and pMPK4) and total MPK6 expression level in wild-type seedlings exposed to 30 µM C10-HSL or Control for 0–60 min. **(d)** Relative expression of pMPK6 in wild type seedlings treated with or without 30 µM C10-HSL for 0–60 min. Control or Con refers to solvent control. All the error bars represent +/- SD (Different letters indicate significantly different values, *P* < 0.05 by Tukey’s test)

Previous studies have shown that MPK3/6 participate in the induction of the defense response by 3OC14-HSL in *Arabidopsis* [9], and that MPK4 regulates primary and lateral root growth [47, 48]. To investigate whether MAPKs play roles in the C10-HSL-induced inhibition of primary root growth, we first assessed the effects of U0126, a selective inhibitor of MAPK, on primary root elongation in wild-type *Arabidopsis* (Columbia-0). Addition of U0126 alleviated the C10-HSL-induced inhibition of primary root growth (Fig. 4a, b) and this effect was dose-dependent (Fig. S3a, b). Western blotting using an anti-phosphate antibody showed that, compared with wild-type seedlings in the control, wild-type seedlings treated with C10-HSL had stronger phosphorylation activity of MPK6, which started at about 5 min and peaked at 45 min after C10-HSL treatment (Fig. 4c, d and Fig. S5 a-d). We also found that MPK3 and MPK4 in *Arabidopsis* were weakly activated from 30 to 60 min after treatment with 30 µM C10-HSL (Fig. 4c, d).

To confirm the involvement of MAPK in the response to C10-HSL, the effects of C10-HSL on primary root growth were compared between wild-type and *mpk6 Arabidopsis* seedlings. The inhibitory effect of C10-HSL on primary root growth was significantly alleviated in the *mpk6* mutant compared with the wild-type seedlings (Fig. 4a, b). Taken together, these results indicate that MAPKs, especially MPK6, are involved in the inhibition of primary root growth modulated by C10-HSL.

### MPK6 mediates the C10-HSL-induced inhibition of primary root growth downstream of ROS and upstream of NO

**Fig. 5:**
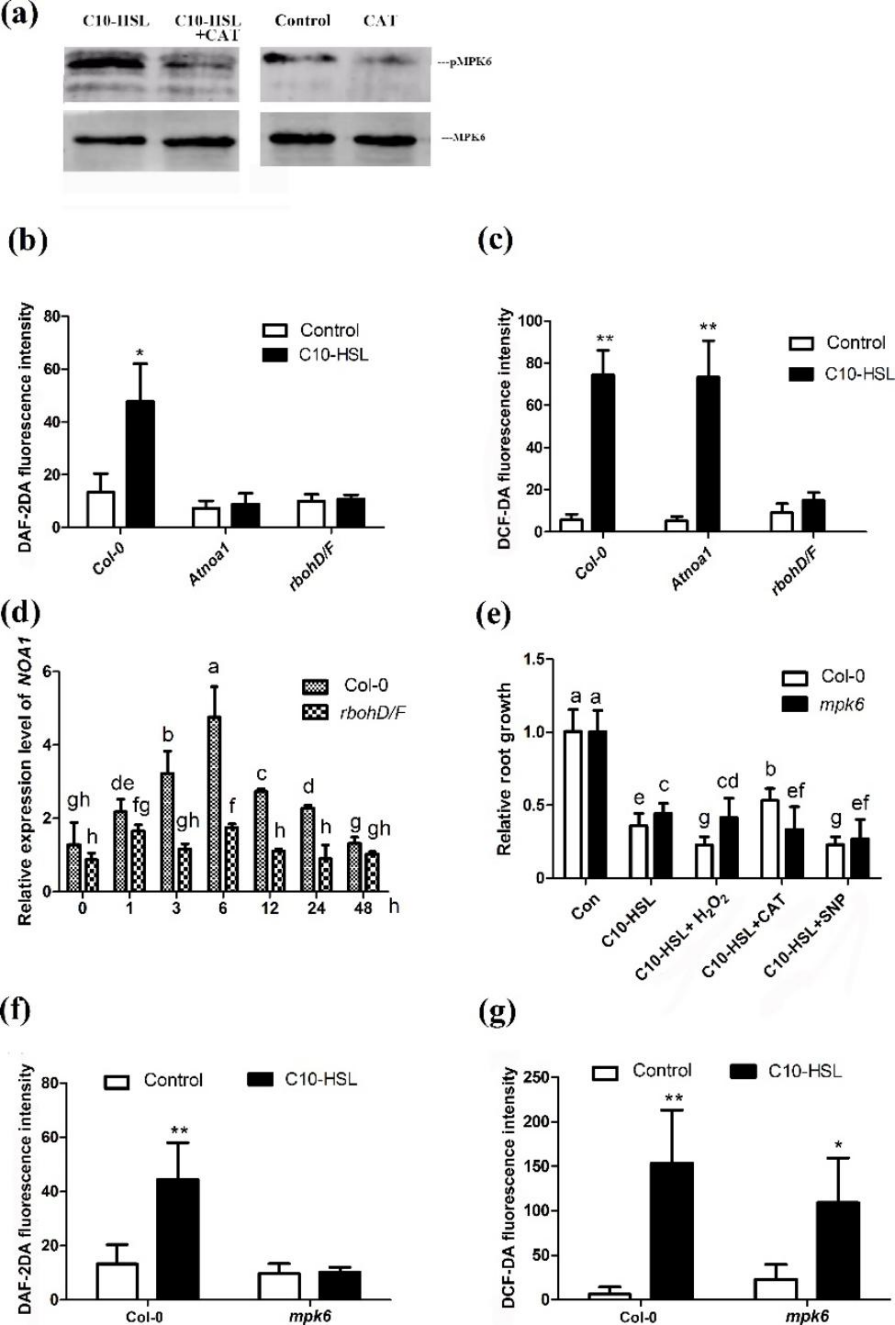
MPK6 mediates the C10-HSL-induced inhibition of primary root growth downstream of ROS and upstream of NO. **(a)** Phosphorylation status (pMPK6) and total expression level of MPK6 in wild-type seedlings exposed to 30 µM C10-HSL with or without 500 µM CAT for 45 min. **(b)** Quantification of NO-specific fluorescence intensities in the roots of Col-0, *rbohD/F* and *noa1* seedlings exposed to 30 µM C10-HSL 1 h using the NO specific florescent probe DAF-2DA. **(c)** Quantification of ROS-specific fluorescence intensities in the roots of Col-0, *rbohD/F* and *noa1* seedlings exposed to 30 µM C10-HSL 15 min using the ROS specific florescent probe DCF-DA. **(d)** Transcriptional regulation of *NOA1* gene after exposed to 30 µM C10-HSL for up to 2 day in Col-0 and *rbohD/F*. **(e)** Relative root growth of Col-0 and *mpk6* seedlings exposed to 30 µM C10-HSL with or without 30 µM SNP, 1.5 mM H_2_O_2_ and 500 µM CAT for 5 d compared with untreated seedlings, n = 40. **(f)** Quantification of NO-specific fluorescence intensities in the roots of Col-0 and *mpk6* seedlings exposed to 30 µM C10-HSL 1 h using the NO specific florescent probe DAF-2DA. (**g)** Quantification of ROS-specific fluorescence intensities in the roots of Col-0 and *mpk6* seedlings exposed to 30 µM C10-HSL 15 min using the ROS specific florescent probe DCF-DA. Control or Con refers to solvent control. All the error bars represent +/- SD. (Different letters indicate significantly different values, *P* < 0.05 by Tukey’s test)

On the basis of the data presented above, we concluded that ROS, NO, and MPK6 are important intermediate components in the regulation of primary root growth by C10-HSL. To explore the interplay among these components, we first detected the effect of the ROS scavenger CAT on the activation of MPK6 induced by C10-HSL. We found that the addition of CAT significantly decreased the activation of MPK6 by C10-HSL (Fig. 5a and Fig. S6 a-c). Next, we measured ROS and NO production in the mutants *noa1* and *rbohD/F*, respectively, after C10-HSL treatment. We found that C10-HSL-induced NO production was abolished in the *rbohD/F* mutant, while C10-HSL-induced ROS production was similar between wild-type and *noa1* (Fig. 5b, c). In addition, the ROS production induced by C10-HSL treatment was unaffected in the *nia1* and *nia2* single mutants and in the *nia1/2* double mutant (Fig. S2d). Further, qRT-PCR analyses showed that the induction of *noa1* expression by C10-HSL in wild-type plants was suppressed in mutant *rbohD/F* seedlings after exposure to C10-HSL (Fig. 5d). These data suggest that C10-HSL induces ROS production upstream of MAPK6 activation and NO accumulation in *Arabidopsi*s roots.

We further checked the NO and ROS production in roots of *Arabidopsis mpk6* mutant seedlings. The induction of NO production by C10-HSL in wild-type seedlings was completely abolished in *mpk6* seedlings (Fig. 5f). In contrast, ROS accumulation in response to C10-HSL treatment was comparable between *mpk6* and wild-type plants (Fig. 5 g). Supplementation with CAT alleviated the C10-HSL-induced inhibition of primary root growth in wild-type roots, but did not further alleviate the C10-HSL-induced inhibition of primary root growth in the *mpk6* mutant (Fig. 5e). In contrast, supplementation with exogenous H_2_O_2_ exacerbated the C10-HSL-mediated inhibition of primary root growth in wild-type seedlings, but did not further increase the inhibitory effects of C10-HSL on primary root growth in the *mpk6* mutant (Fig. 5e). Supplementation with the NO donor sodium nitroprusside (SNP) increased the inhibitory effects of C10-HSL on the primary root growth of both *mpk6* and wild-type seedlings (Fig. 5e). Considering all these results, we concluded that MPK6 mediates the C10-HSL-induced inhibition of primary root growth downstream of ROS and upstream of NO.

### C10-HSL triggers an elevated concentration of intracellular calcium and controls ROS and NO accumulation

**Fig. 6:**
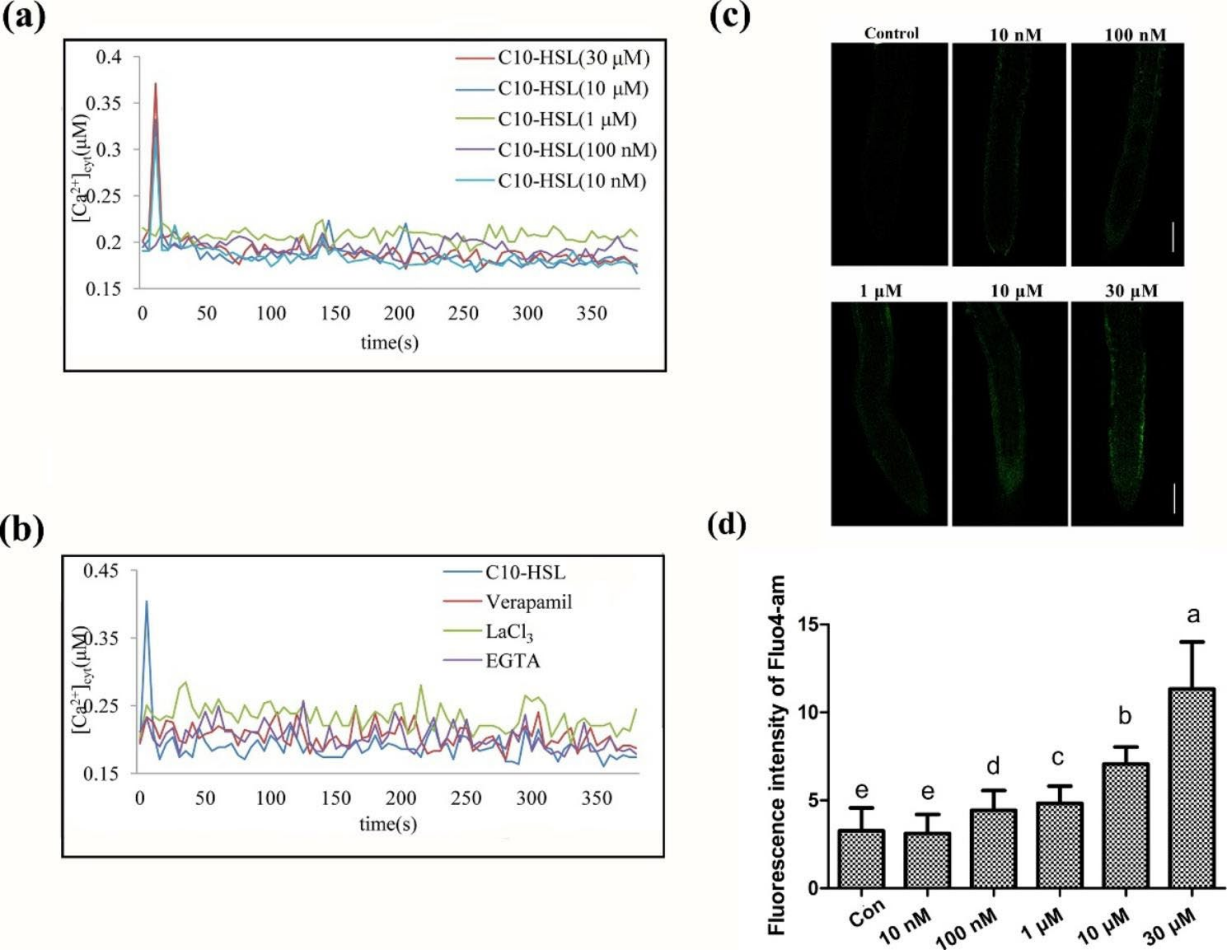
C10-HSL improved cytosolic Ca^2+^ concentration in wild-type seedlings. **(a)** Changes in [Ca^2+^]_cyt_ in apoaequorin-transformed Arabidopsis root cells with 10 nM, 100 nM, 1 µM, 10 µM, 30 µM C10-HSL. **(b)** Changes in [Ca^2+^]_cyt_ in Arabidopsis root cells were measured upon addition of 30 µM C10-HSL after pretreatment with LaCl_3_, EGTA and Verapamil respectively. **(c, d)** Detection of cytosolic Ca^2+^ concentration in the roots of Col-0 seedlings exposed to 0 nM, 10 nM, 100 nM, 1µM, 10 µM and 30 µM C10-HSL using the Ca^2+^ specific florescent probe Fluo4-am, bar = 100 μm. Control or Con refers to solvent control. All the error bars represent +/- SD. (Different letters indicate significantly different values, *P* < 0.05 by Tukey’s test)

**Fig. 7:**
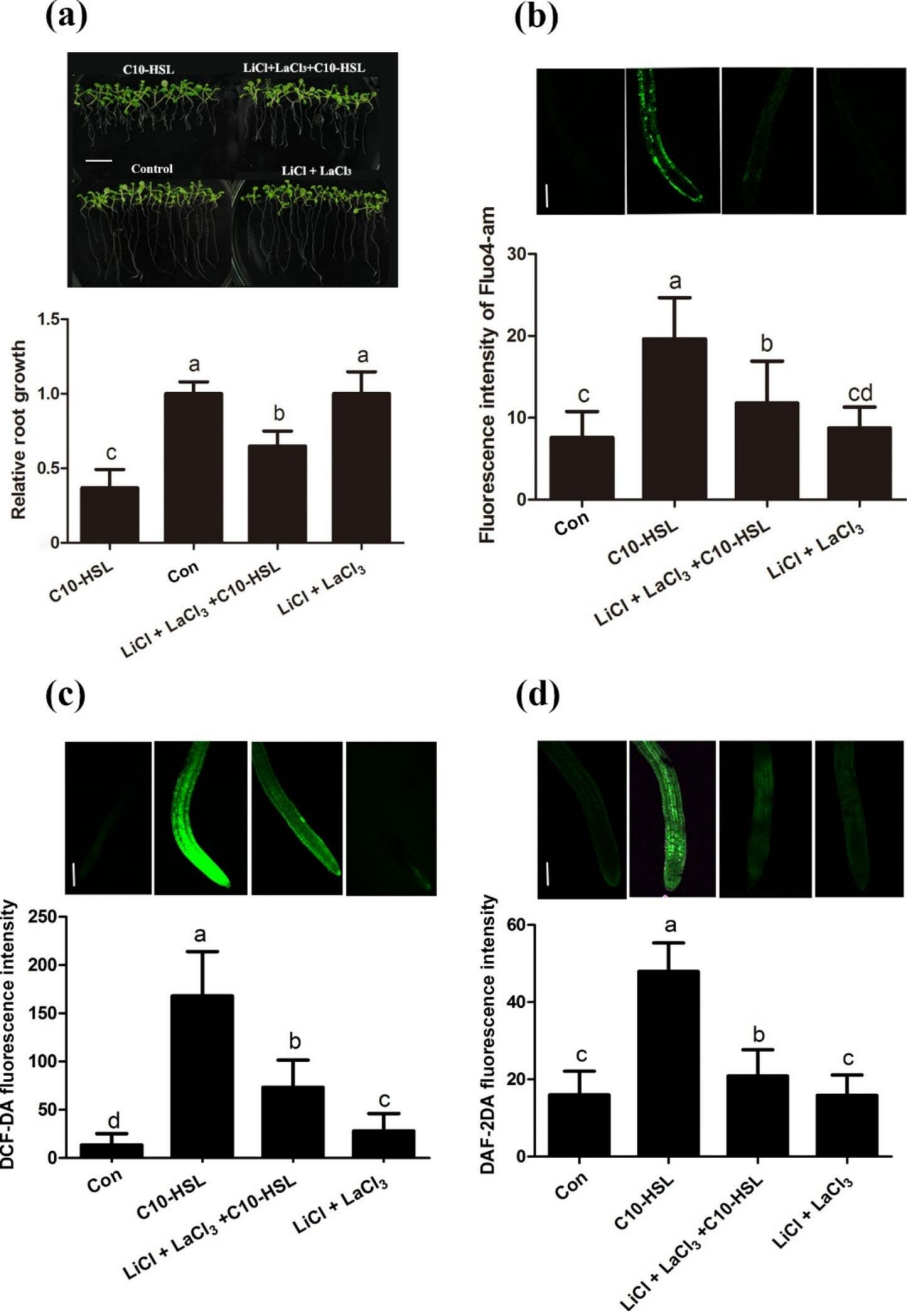
Up-regulated cytosolic Ca^2+^ induced ROS and NO reaction which is necessary for C10-HSL induced root growth inhibition. **(a)** Relative primary root growth of Col-0 seedlings exposed to 30 µM C10-HSL with or without 100 µM LiCl plus 300 µM LaCl_3_ compared with control for 5 d, bar = 1 cm, n = 40. **(b)** Detection of cytosolic Ca^2+^ concentration in the roots of Col-0 seedlings exposed to 30 µM C10-HSL with or without 100 µM LiCl plus LaCl_3_ compared with untreated seedlings using the Ca^2+^ specific florescent probe Fluo4-am and quantification of Ca^2+^-specific fluorescence intensities, bar = 100 μm, n = 30. **(c)** Detection of ROS production in the roots of Col-0 seedlings exposed to 30 µM C10-HSL with or without 100 µM LiCl plus LaCl_3_ for 15 min using the specific florescent probe DCF-DA and quantification of specific fluorescence intensities, bar = 100 μm, n = 30. **(d)** Detection of NO production in the roots of Col-0 seedlings exposed to 30 µM C10-HSL with or without 100 µM LiCl plus LaCl_3_ for 15 min using the NO specific florescent probe DAF-2DA and quantification of NO-specific fluorescence intensities, bar = 100 μm, n = 30. Control or Con refers to solvent control. All the error bars represent +/- SD. (Different letters indicate significantly different values, *P* < 0.05 by Tukey’s test)

Cytosolic Ca^2+^ is a second messenger that plays an essential role in plant root growth [49, 50]. The effect of C10-HSL on [Ca^2+^]_cyt_ was evaluated in root cells of transgenic *Arabidopsis* expressing the gene encoding intracellular apoaequorin. The results showed that the [Ca^2+^]_cyt_ level in *Arabidopsis* root cells significantly increased within 30 s of adding C10-HSL at concentrations from 10 nM to 30 µM (Fig. 6a). Pretreatments with La^3+^, verapamil, or EGTA blocked the elevation in [Ca^2+^]_cyt_ caused by 30 µM C10-HSL (Fig. 6b). To further monitor whether C10-HSL affects the level of [Ca^2+^]_cyt_ in root cells, the fluorescence of the Ca^2+^-sensitive dye Fluo-4 AM was analyzed by confocal microscopy. The fluorescence intensity level significantly increased after exposure to C10-HSL at concentrations higher than 100 nM in a dose-dependent manner (Fig. 6c, d). These data suggested that C10-HSL immediately triggers an elevation of [Ca^2+^]_cyt_ in *Arabidopsis* root cells. LiCl, a known inhibitor of the phosphatidylinositol cycle, inhibits the release of Ca^2+^ from intracellular calcium pools, while LaCl_3_ inhibits the import of Ca^2+^ across the plasma membrane. Addition of both these compounds to the medium containing C10-HSL buffered its inhibitory effect on primary root growth in wild-type seedlings (Fig. 7a and Fig S4), implying that Ca^2+^ plays a role in C10-HSL-induced inhibition of primary root growth in *Arabidopsis.* To investigate whether Ca^2+^ affects ROS and NO production induced by C10-HSL, we measured the fluorescence intensity of DCF-DA as well as DAF-2DA and found that the addition of Ca^2+^ inhibitors down-regulated both ROS and NO production induced by C10-HSL, consistent with the decrease in [Ca^2+^]_cyt_ detected by Fluo-4 AM analyses (Fig. 7b–d). Taken together, these results indicate that C10-HSL increases the intracellular calcium concentration and controls the production of both ROS and NO.

## Discussion

Many Gram-negative bacteria can use AHLs as intracellular signals to monitor their population density by QS [1–3]. AHLs not only play an essential role in QS-mediated physiological processes, but also modulate plant growth, defense responses, and development [11, 17, 18, 51–53]. C10-HSL or 3OC10-HSL can be produced by several bacteria including the rhizosphere-colonizing bacteria *Pseudomonas fluorescens* and *Pseudomonas putida* [54], the nitrogen-fixing bacterial symbiont *Sinerhizobium meliloti* [55], and the pathogenic bacteria *Burkholderia pseudomallei* and *Burkholderia mallei* [2]. The concentration of AHLs in natural niches is estimated to range from 1 nM to 10 µM in the bulk rhizosphere, although the concentrations can be higher (> 50 µM) in bacterial biofilms [56, 57]. Ortíz-Castro et al. (2008) tested the effects of seven AHLs with concentrations ranging from 12 to 192 µM on root growth, and found that C10-HSL showed the strongest effect to inhibit primary root growth and stimulate lateral root formation and root hair development [18]. A derivative of C10-HSL, 3OC10-HSL, was found to stimulate adventitious root formation in mung bean [11]. In this study, we confirmed the effect of C10-HSL and showed that treatment with 10 nM to 1 µM C10-HSL significantly reduced primary root growth and promoted lateral root formation. The effect of C10-HSL on root system architecture was concentration-dependent, and 30 µM C10-HSL caused a 74% reduction in primary root length that was accompanied by 3.5-fold increase in lateral root formation. These results were consistent with those reported by Bai et al. (2012) [11] and Ortíz-Castro et al. (2008) [18], and confirm that AHLs can mediate cross-kingdom interactions by modulating root development during the plant-bacteria interaction. Here, we focused on primary root growth and chose 30 µM C10-HSL as the effective concentration for further analyses. This concentration is somewhat higher than that produced by bacteria in the bulk rhizosphere but comparable to that present in bacterial biofilms.

In plants, ROS are involved in the responses to various phytohormones and environmental cues. We found that treatment with 30 µM C10-HSL increased ROS accumulation in roots, and this led to inhibition of primary root growth in *Arabidopsis*. The inhibitory effect of C10-HSL on primary root growth was alleviated by supplementation with CAT, but exaggerated by exogenous H_2_O_2_. This indicated that H_2_O_2_, one component of ROS, contributes to C10-HSL-mediated primary root growth inhibition in *Arabidopsis*. Further evidence was obtained from genetic analyses of the ROS-related mutant *rbohD/F*. We found that the primary roots of *rbohD/F* seedlings were less sensitive to C10-HSL treatment. Zhang et al. (2017) reported that ROS generation is required for exogenous H_2_S-induced inhibition of primary root growth in *Arabidopsis* [27]. Similarly, ROS were found to accumulate in the root tips after treatment with the nitrification inhibitor methyl 3-(4-hydroxyphenyl) propionate (MHPP), which inhibited primary root elongation in *Arabidopsis* [58]. These data show that AHLs influence primary root growth by regulating ROS accumulation in plant roots.

Another important signaling molecule is NO, which regulates root growth in plants. As AHL homologs, plant alkamides were found to induce accumulation of NO and promote adventitious root development in *Arabidopsis thaliana* explants [59]. Pharmacological analyses indicated that blocking the 3OC8-HSL-induced accumulation of NO with the NO scavenger cPTIO significantly reduced the effect of 3OC10-HSL to stimulate adventitious root formation in mung bean, suggesting a role of NO in 3OC10-HSL-mediated root development [11]. In our experiments, we found that C10-HSL triggered NO accumulation in root tips. Moreover, supplementation with an NO scavenger abolished the production of NO induced by C10-HSL, and repressed the inhibitory effects of C10-HSL on primary root growth. Furthermore, we found that *NOA1 and NIA* were upregulated by C10-HSL, and that C10-HSL-mediated primary root growth inhibition was markedly impaired in the *NOA1*-defective mutant *noa1 and NIA* mutant *nia1nia2*. These results provide pharmacological and genetic evidence that NO is required for C10-HSL-inhibited primary root growth in *Arabidopsis*. Heavy metals such as zinc and cadmium and toxic chemicals including H_2_S and MHPP inhibit primary root growth by increasing the concentration of intracellular NO, suggesting that NO is also involved in inhibiting primary root growth in response to environmental factors [27, 58].

In plants, MAPKs are activated in response to a number of environmental cues [60–62]. In this study, the MAPK inhibitor U0126 neutralized the C10-HSL-induced inhibition of primary root growth, and C10-HSL treatment strongly activated MPK6 and weakly activated MPK3 and MPK4 within 30 min. These findings indicate that activation of MAPK is required for the inhibition of primary root growth by C10-HSL. Consistent with this result, an MPK6 mutant showed reduced inhibition of primary root growth after C10-HSL treatment, providing molecular evidence for the essential role of MPK6 in C10-HSL-mediated inhibition of primary root growth. Previously, Schikora et al. (2011) reported that application of 3OC14-HSL induces resistance against bacterial and fungal pathogens, and this effect depends on strong and prolonged activation of MPK6 in *Arabidopsis* [9]. Accordingly, these data show that MPK6 is required for AHL-mediated changes in primary root growth.

MPK6 modulates plant growth and the response to environmental stimuli by interacting with ROS and/or NO [36, 37, 63, 64]. In *Arabidopsis*, NO stimulates cadmium-induced programmed cell death by enhancing MPK6-mediated caspase-3-like activation [65]. Compared with wild-type, the MPK6 mutant formed more and longer lateral roots after application of SNP but H_2_O_2_, indicating that MPK6 modulates NO accumulation and responses to H_2_O_2_ during root development in *Arabidopsis* [63]. Liu et al. (2016) reported that NO/ROS accumulation contributes to MHPP-mediated primary root growth inhibition and NO acts upstream of ROS in the response to MHPP in *Arabidopsis* [58]. Using pharmacological approaches, Bai et al. (2012) showed that H_2_O_2_ may modulate the NO signal during the response to 3OC10-HSL treatment in mung bean [11]. The results of those studies suggest that MPK6, ROS, and NO interact, but the mechanism underlying this interaction may be context-dependent. In this study, we observed that reducing ROS accumulation with the H_2_O_2_ scavenger CAT significantly decreased the activation of MPK6. We also found that C10-HSL-induced NO production was abolished in the *rbohD/F* mutant, while C10-HSL-induced accumulation of ROS was unaffected in the *noa1* mutant. In addition, C10-HSL treatment did not upregulate *NOA1* in the *rbohD/F* mutant. The induction of NO production by C10-HSL was completely abolished in the *mpk6* mutant, while ROS accumulation caused by C10-HSL treatment was unaffected. Addition of CAT did not further alleviate the C10-HSL-induced inhibition of primary root growth in roots of the *mpk6* mutant. In contrast, the defect in the C10-HSL-induced inhibition of primary root growth in the *mpk6* mutant was rescued by application of SNP, an NO donor, but not by addition of exogenous H_2_O_2_. Our data indicate that ROS, MPK6, and NO might work together to regulate plant root responses to C10-HSL. A similar signaling pathway has been reported to participate in the plant response to H_2_S toxicity [27].

In plants, Ca^2+^ functions as an intracellular second messenger, and is essential for signal transduction processes [40, 41]. In our previous study, we showed that C4-HSL triggered an increase in the concentration of intracellular Ca^2+^ [9]. In the present study, we found that treatment with C10-HSL resulted in a transient and immediate increase in [Ca^2+^]_cyt_ in root cells of *Arabidopsis*. The effect of C10-HSL to increase the [Ca^2+^]_cyt_ concentration was investigated using the Ca^2+^-sensitive dye Fluo-4 AM. These analyses showed that the inhibition of the increase in the intracellular Ca^2+^ concentration reduced the inhibitory effect of C10-HSL on primary root growth. Moreover, the addition of a Ca^2+^ inhibitor significantly reduced the production of ROS and NO induced by C10-HSL. Our data indicate that Ca^2+^ signaling may be an early event in the plant response to C10-HSL, and it may regulate the down-stream production of ROS. These mechanisms also occur during incompatible host-pathogen recognition, when the flux of Ca^2+^ across the plasma membrane is one of the earliest cellular events and results in a set of oxidative bursts that produce ROS [66, 67].

**Fig. 8:**
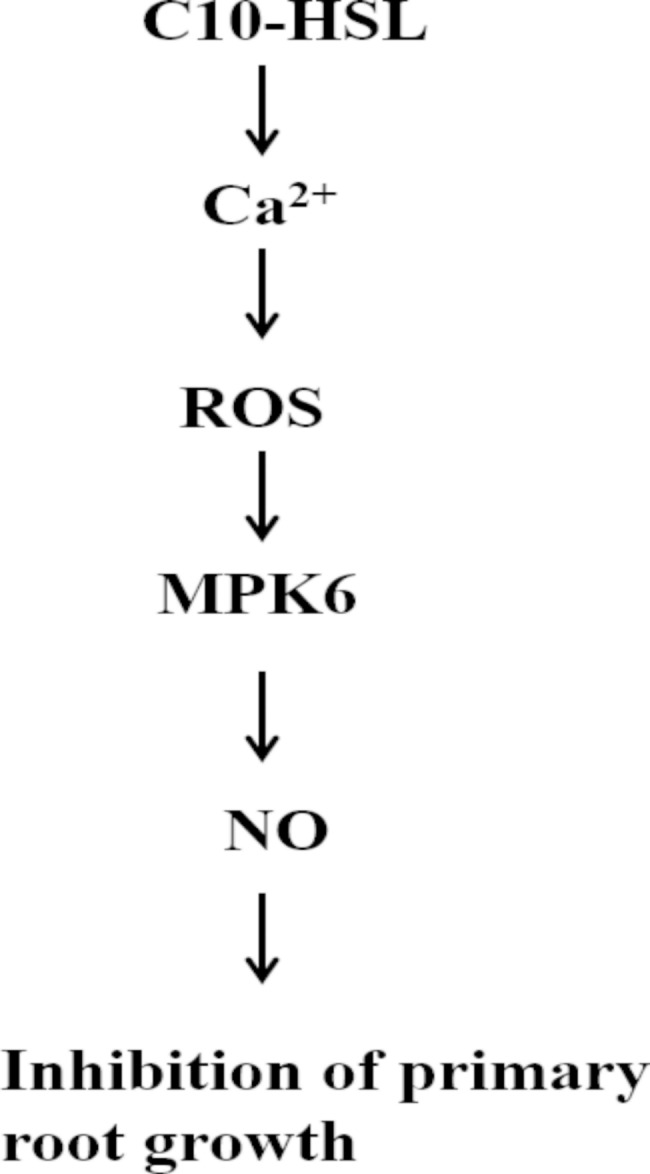
The tentative model for C10-HSL-induced inhibition of primary root growth. C10-HSL triggered cytosolic free Ca^2+^ and reactive oxygen species (ROS), increased the activity of mitogen-activated protein kinase 6 (MPK6), and induced nitric oxide (NO) production in *Arabidopsis*, and finally inhibited primary root growth

In conclusion, our results show that C10-HSL triggered a transient and immediate increase in the concentration of cytosolic free Ca^2+^ and induced ROS accumulation, MPK6 activation, and NO production in *Arabidopsis* primary roots. The generation of ROS and NO induced by C10-HSL was mediated by Ca^2+^. Activation of MPKs was necessary for the inhibition of primary root growth by C10-HSL. MPK6 was shown to act downstream of ROS and upstream of NO. Thus, our data suggest that Ca^2+^, ROS, MPK6, and NO are all involved and might play roles in cascade in the C10-HSL-mediated inhibition of primary root growth in *Arabidopsis* (Fig. 8). Auxin plays a fundamental role in root system architecture in plants [68, 69]. However, Ortiz-Castro et al. (2008) pointed out that C10-HSL is independent of auxin signaling in *Arabidopsis*, although the C10-HSL-mediated inhibition of primary root growth is similar to the typical auxin signaling phenotype [18]. However, 3OC12-HSL and 3OC16-HSL were found to induce the tissue-specific expression of auxin-responsive *GH3* in legume [13]. Bai et al. (2012) reported that 3OC10-HSL, an analog of C10-HSL, was able to mediate auxin-dependent adventitious root formation *via* H_2_O_2_- and NO-dependent cGMP signaling in mung bean seedlings [11]. C6-HSL was found to promote root elongation and increase the ratio of auxin: cytokinin towards higher auxin levels in both leaves and roots of *Arabidopsis* [51]. Zhang et al. (2017) reported that exogenous H_2_S repressed the distribution of auxin and inhibited primary root growth in *Arabidopsis* [27]. The nitrification inhibitor MHPP was found to modulate root system architecture *via* ROS/NO-mediated-accumulation and redistribution of auxin in *Arabidopsis* roots [60]. Moreover, Hu et al. (2018) found that *N*-decanoyl-homoserine lactone (C10-HSL) activated plant systemic resistance against *Botrytis cinerea* in tomato and C10-HSL-induced resistance was largely dependent on the JA signaling pathway [19]. Recently, we demonstrated that 3OC8-HSL primes plant resistance against necrotrophic pathogen *Pectobacterium carotovorum* by coordinating JA and auxin signaling pathway [70]. Therefore, comprehensive studies on the roles of phytohormones such as auxin and JA and their interplay with C10-HSL will shed light on the mechanism by which plants respond to C10-HSL, and will provide insight into novel applications of these biological molecules to regulate crop growth and development.

## Materials and methods

### Plant growth and chemical treatments

The *Arabidopsis thaliana* ecotype Columbia-0 (Col-0), the mutants *Atmpk6* (obtained from the TAIR stock center), *nia1*, *nia2*, *noa1* (provided by Professor Chunpeng Song in Henan University, China), *rbohD/F* (provided by Professor Zhonglin Shang in Hebei Normal University, China) were used in this study. Seeds were surface sterilized with 75% ethanol for 1 min and 25% bleach for 5 min. After five washes with sterile distilled water, seeds were germinated on agar plates containing half-strength Murashige & Skoog (MS) medium [71] adjusted to pH 5.8. The plates were stratified at 4 °C for 2 days and then placed in a plant growth chamber with a photoperiod of 16 h: 8 h light: dark with 100 µmol·m^2^·s^− 1^ and temperature of 22 ± 2 °C. Seeds were grown on the vertical position. 3-d-old *Arabidopsis thaliana* seedlings were transferred to half-strength MS medium containing supplemented with various chemicals, including 30 µM C10-HSL, sodium nitroprusside (SNP), 2-(4-carboxyphenyl)-4,4,5,5-tetramethylimidazoline-1-oxyl-3-oxide (cPTIO), hydrogen peroxide (H_2_O_2_), catalase (CAT), U0126, lithium chloride (LiCl) and lanthanum chloride (LaCl_3_) and grown for additional 5 d. All chemicals were purchased from Sigma-Aldrich and C10-HSL was dissolved in ethanol as 30 mM stock solutions.

### Analysis of root growth

Seeds were grown on the vertical position. 3-d-old *Arabidopsis thaliana* seedlings were transferred to half-strength MS medium supplemented with the indicated treatments and grown for additional 5 d. Growth of primary roots was measured at the same time every day. After 5 d of treatment, the primary root length was measured using a ruler. Lateral root (LR) number and lateral root primordia (LRP) were determined by counting the lateral roots or LRP present in the primary root, from the tip to the root/stem transition. Lateral root and primordia densities were determined by dividing the lateral root number by the primary root length and expressed as LR cm^− 1^ or LRP cm^− 1^. The stage C-D of lateral root primordia according to Zhang et al. (1999) [72] were considered for the research. All experiments were performed with the solvent (ethanol) as a control at an end dilution same as AHL tested.

### Immuno-detection of phosphorylated MAPKs

According to the method mentioned by Schikora et al. (2011) [9], total protein was extracted from 3-d-old seedlings pretreated with C10-HSL in half-strength MS medium for additional 5, 15, 30, 45 and 60 min. The total proteins were separated on 12% SDS polyacryl gel. Western blot was done using Phospho-p44/42 MAPK antibody purchased from Cell signaling (CAT. #4370) [9]. Licor-ODYSSEY(CLX) was used to photo blotting images. To obtain higher definition, we used a drawing area function supported by the machine.

### Dye loading and laser scanning confocal microscopy

The generation of NO, ROS and Ca^2+^ in root tissue was examined using 4, 5-diaminofluorescein diacetate (DAF-2DA) (Sigma Aldrich), 5-(and-6)-chloromethyl-29, 79-dichlorodihydrofluorescein diacetate (DCF-DA) (Sigma Aldrich) and Fluo-4 AM (Thermo) probes, respectively. Before experiments, roots of seedlings were pre-incubated in the dark in loading buffer (10 mM Tris + 50 mM KCL, pH 7.18) containing 5 µM DAF-2DA or 10 µM H2DCF-DA for 30 min, or in buffer (50 mM KCL + 10 mM Tris + 5 mM CaCL_2_ + 0.5 mM eserine, pH 7.18) containing 10 µM Fluo-4 AM for 1 h, and then washed with distilled water to remove excess dye. Examinations of fluorescence intensity were performed using a laser scanning confocal microscope (excitation, 488 nm; emission, 500–550 nm; Leica SP8). For the fluorescence of dyes and GFP, we measured the fluorescence value of all meristematic zone in every plant, using internal Quantity software of Leica SP8 confocal microscope.

### RT-qPCR

Total RNA was isolated from root tissues of 12-d-old seedlings treated with the indicated reagents for 0, 1, 3, 6, 12, 24 and 48 h, using Trizol reagent (Invitrogen, Carlsbad, CA, USA). First-strand cDNA was synthesized using Moloney murine leukaemia virus reverse transcriptase (Invitrogen). PCR amplifications were performed according to standard protocols using Taq DNA polymerase or Pyrobest DNA polymerase (TaKaRa). The mean value of three replicates was normalized against that of *ACTIN2*.

### [Ca ^2+^]_cyt_ measurements

Arabidopsis seedlings constitutively expressing the aequorin gene were germinated and grown on the half-strength MS agar plates at 23 °C and a 10 h dark/14 h light regime for 10 days. The [Ca^2+^]_cyt_ rises either as a result of uptake from the extra-cellular space through plasma membrane channels or from the release of internal stores. Roots were incubated in 10 µM coelenterazine (0.1 mM KCL, 1 mM CaCL_2_,10 mM MES, pH 5.5) for 24 h and rinsed with buffer for 30 min. Each seedling was placed in a luminometer cuvette containing 50 µl buffer solution. C10-HSL stock solution was then injected (50 µl per injection) to achieve the indicated concentration. Final concentrations of LaCL_3_, verapamil chloride and EGTA were 10 mM, respectively. Thirty pre-washed seedlings were used for each luminometric experiment. Real-time aequorin luminescence was recorded every 1 s over the course of the experiment. At the end of the experiment, 2 M CaCl_2_ and 10% ethanol were added to discharge any remaining aequorin.

## Electronic supplementary material

Below is the link to the electronic supplementary material.

Supplementary Material 1: Figure S1. Primary root length and lateral root density of wild-type seedlings exposed to C10-HSL. Figure S2. NIA1, NIA2 and NOA1 participate in C10-HSL induced short primary root. Figure S3. U0126 could rescue C10-HSL induced primary root inhibition. Figure S4. Relative primary root growth of Col-0 seedlings exposed to C10-HSL with or without LiCl plus LaCl3. Table 1. List of the primers for qRT-PCR analysis of the genes. Bloting images (Figure S5 and S6): Original blotting images.

## Data Availability

The datasets used and/or analyzed during the current study are available from the corresponding author on request.

## Declarations

### Author details

^1^ Biology Institute, Hebei Academey of Sciences, 46th South Street of Friendship, Shijiazhuang 050051, Hebei, China. ^2^ Hebei Engineering and Technology Center of Microbiological Control on Main Crop Disease, 46th South Street of Friendship, Shijiazhuang, China.^3^ College of Life Science, Hebei University, 180th East Road of Wusi, Baoding, China. ^4^ Hebei Collaboration Innovation Center for Cell Signaling Environmental Adaptation, 20 East NanErhuan Road, Shijiazhuang, China.

## Abbreviations

AHLs: *N*-acyl-homoserine lactones
CAT: catalase C10-HSL:*N*-decanoyl-L-homoserine lactone
cPTIO: 2-(4-carboxyphenyl)-4,4,5,5-tetramethylimidazoline-1-oxyl-3-oxide
C6-HSL: *N*-hexanoyl-homoserine lactone
DAF-2DA: 4, 5-diaminofluorescein diacetate
DCF-DA: 5-(and-6)-chloromethyl-29, 79-dichlorodihydrofluorescein diacetate
GCR: G-protein-coupled receptor
H_2_O_2_: hydrogen peroxide
LaCl_3_: lanthanum chloride
LiCl: lithium chloride
LR: Lateral root
LRP: lateral root primordia
MAPK: Mitogen-activated protein kinase
MPK6: *Mitogen-Activated Protein Kinase 6*
NO: nitric oxide
QS: quorum sensing
ROS: reactive oxygen species
SA: salicylic acid
SNP: sodium nitroprusside
3OC8-HSL: 3-oxo-octanoyl-homoserine lactone
3OC10-HSL: *N*-3-oxo-decanoyl-homoserine lactone
3OC14-HSL: *N*-3-oxo-tetradecanoyl-homoserine-lactone
3OC16-HSL: 3-oxo-*N*-(tetrahydro-2-oxo-3-furaryl)-hexadecenamide
