# Supplementary material for: Cellular messengers involved in the inhibition of the Arabidopsis primary root growth by bacterial quorum-sensing signal N-decanoyl-L-homoserine lactone
Source: BMC Plant Biol. 2022 Oct 14;22:488. doi: 10.1186/s12870-022-03865-6 (PMC9563914; doi:10.1186/s12870-022-03865-6)
Supplement: Supplementary file 1 — Supplementary Material 1: Figure S1. Primary root length and lateral root density of wild-type seedlings exposed to C10-HSL. Figure S2. NIA1, NIA2 and NOA1 participate in C10-HSL induced short primary root. Figure S3. U0126 could rescue C10-HSL induced primary root inhibition. Figure S4. Relative primary root growth of Col-0 seedlings exposed to C10-HSL with or without LiCl plus LaCl3. Table 1. List of the primers for qRT-PCR analysis of the genes. Bloting images (Figure S5 and S6): Original blotting images. [file 12870_2022_3865_MOESM1_ESM.docx]

**Supplimental Data**

**Figure S1**


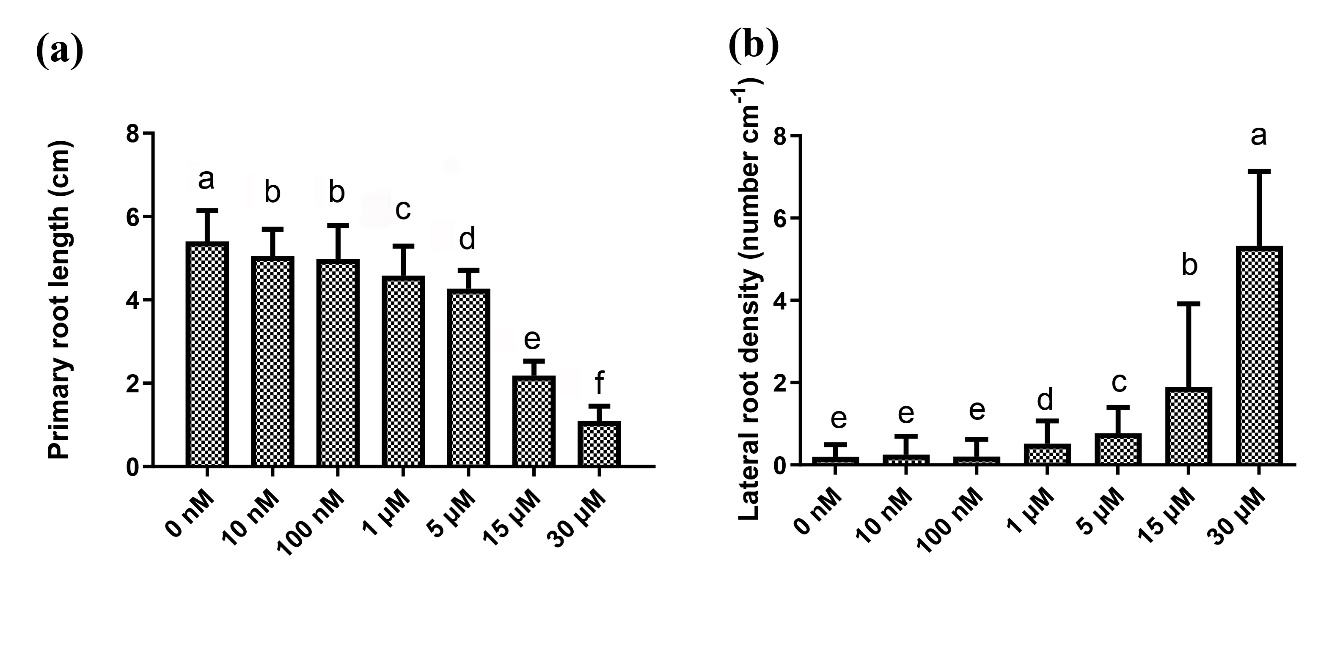


Primary root length (a) and lateral root density (b) of wild-type seedlings exposed to 0 - 30 μM C10-HSL for 5 d, n=40. All the error bars represent +/- SD. (Different letters indicate significantly different values, *P* < 0.05 by Tukey’s test).

**Figure S2**


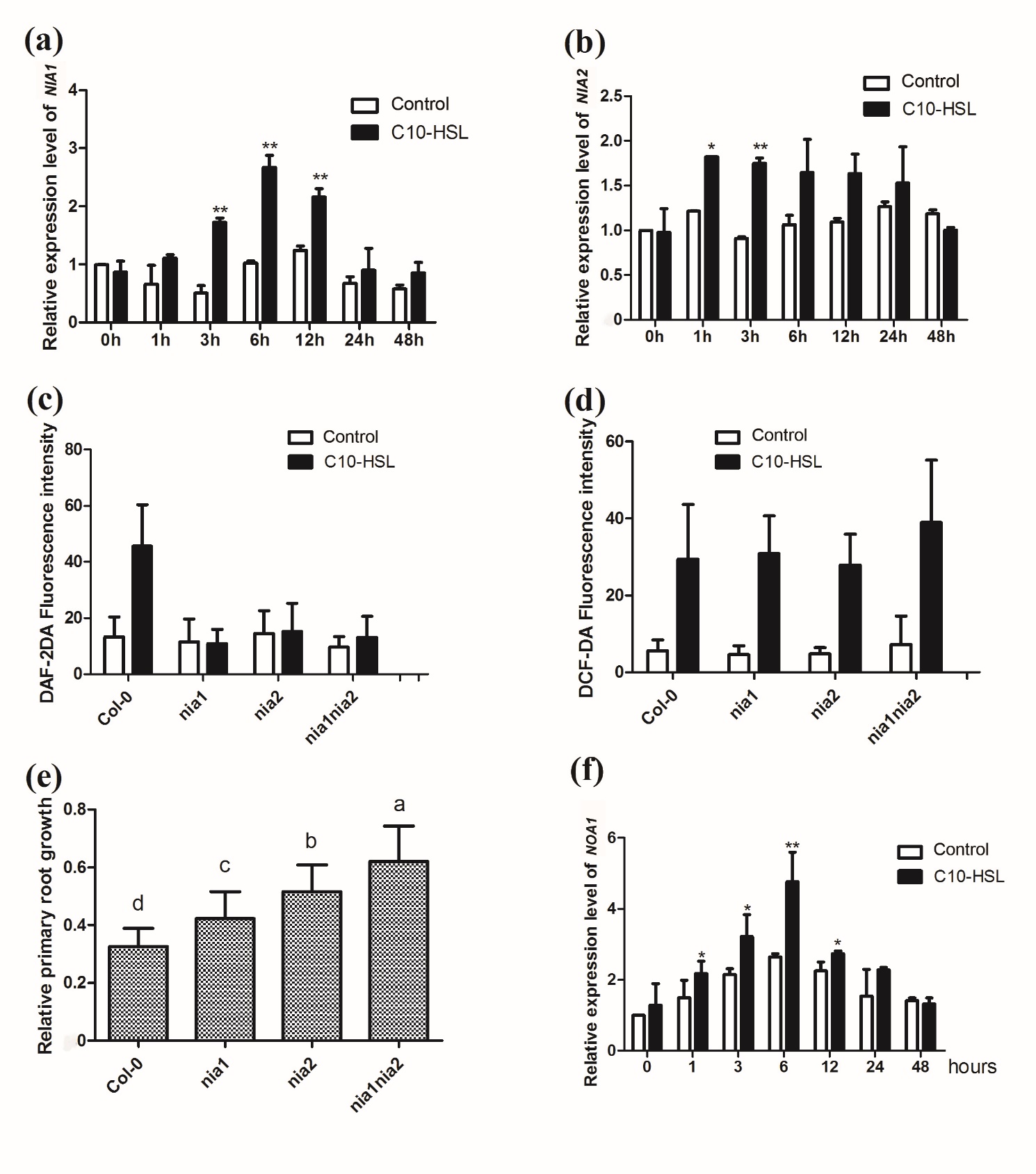


NIA1, NIA2 and NOA1 participate in C10-HSL induced short primary root. **(a, b)** Transcriptional regulation of *NIA1* and *NIA2* genes after exposed to 30μM C10-HSL for up to 2 days in Col-0. **(c, d)**, Detection of NO production(c) and ROS production **(d)** in the roots of Col-0, *nia1, nia2* and *nia1nia2* seedlings exposed to 30 μM C10-HSL for 1 d. **(e)**, Relative primary root growth of Col-0, *nia1, nia2* and *nia1nia2* seedlings exposed to 30μM C10-HSL for 5 d, n>=30. **(f)** Relative expression level of *NOA1* gene after exposure to 30 μM C10-HSL for up to 2 d in wild-type seedlings. Control refers to solvent control. All the error bars represent +/- SD. (Different letters indicate significantly different values, *P* < 0.05 by Tukey’s test; **P* < 0.05, ***P* < 0.001, Student’s t test).

*****

******

******

******

******

**Figure S3**


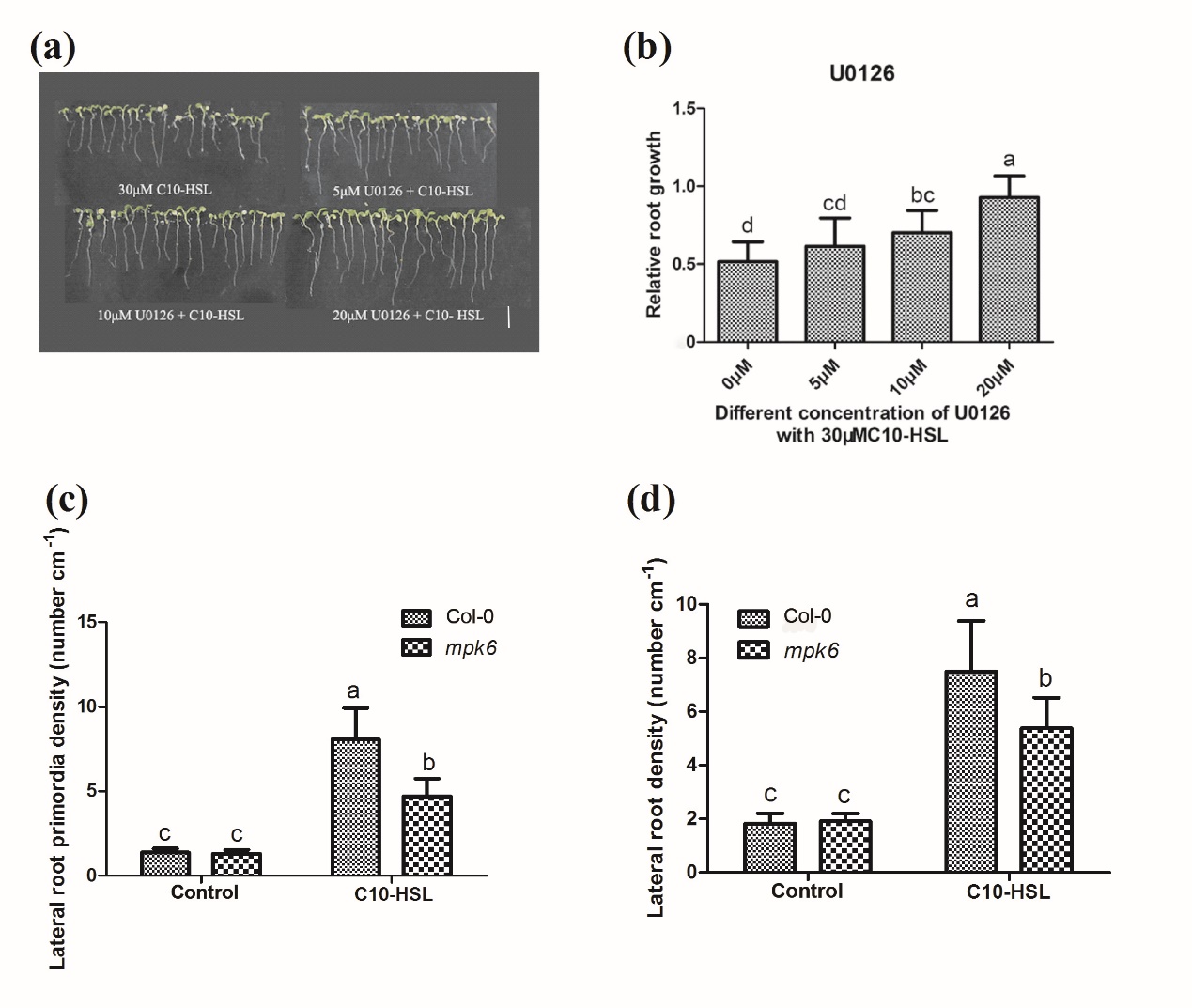


U0126 could rescue C10-HSL induced primary root inhibition. **(a, b)** Relative primary root growth of Col-0 seedlings exposed to 30 μM C10-HSL with or without 5 - 20 μM U0126 for 5 d, n=40, bar=1cm. **(c, d)**, Lateral root density (c) and Lateral root primordia density (d) of wild type seedlings exposed 30μM C10-HSL compared with Control for 5 d. Control refers to solvent control. All the error bars represent +/- SD. (Different letters indicate significantly different values, *P* < 0.05 by Tukey’s test)

**Figure S4**


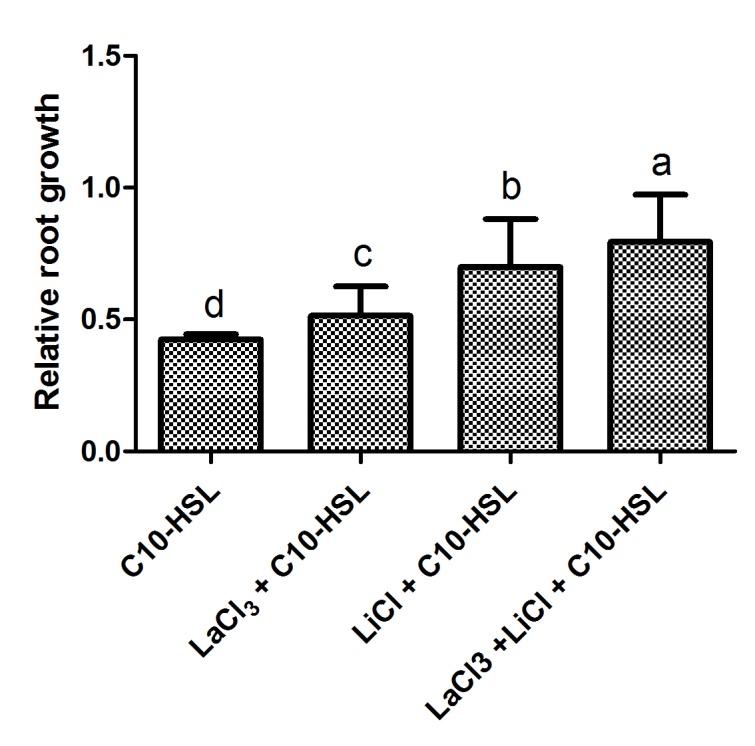


Relative primary root growth of Col-0 seedlings exposed to 30 μM C10-HSL with or without 100 μM LiCl plus 300 μM LaCl_3_ compared for 5 d, n=40. All the error bars represent +/- SD. (Different letters indicate significantly different values , *P* < 0.05 by Tukey’s test)

**Table1. List of the primers for qRT-PCR analysis of the genes.**

| Primer names | Primer sequences (5'-3') |
| --- | --- |
| NOA1-F | TCGGAGGAGAGAAGTTGTATGAC |
| NOA1-R | ATTTTGAGGAGCAAGGGCG |
| NIA1-RT-F | TGAAGGTGGATGGCGAACTC |
| NIA1-RT-R | CGTCCACGGTCACATCT |
| NIA2-RT-F | CCTAAGGGAGGAACTGGA |
| NIA2-RT-R | ACTGAATCATCGGTGGTG |
| ACTIN2-F | GGTAACATTGTGCTCAGTGGTGG |
| ACTIN2-R | AACGACCTTAATCTTCATGCTGC |

**Blotting images**

**Figure S5 (a)**: Phos-MPK6 blotting of C10-HSL treatment for 0-60 min.


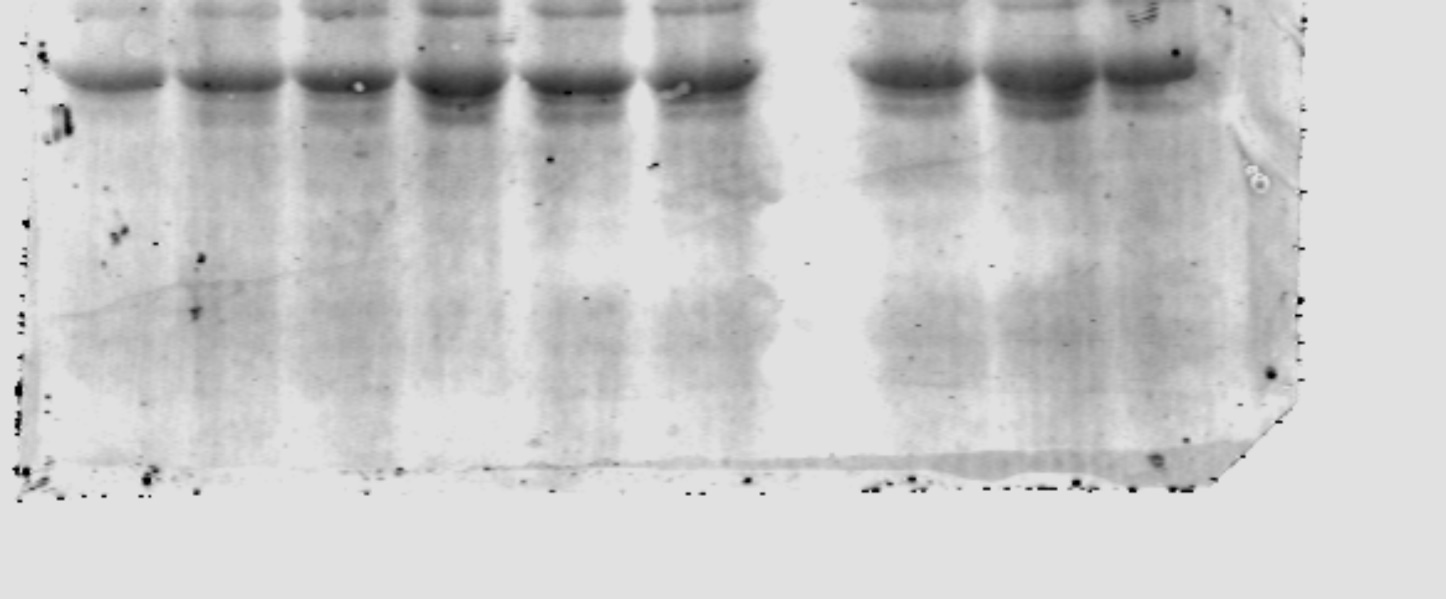


0 5 15 30 45 60（min）

Phos-MP6

**Figure S5 (b)**: Phos-MPK6 blotting of Control treatment for 0-60 min.


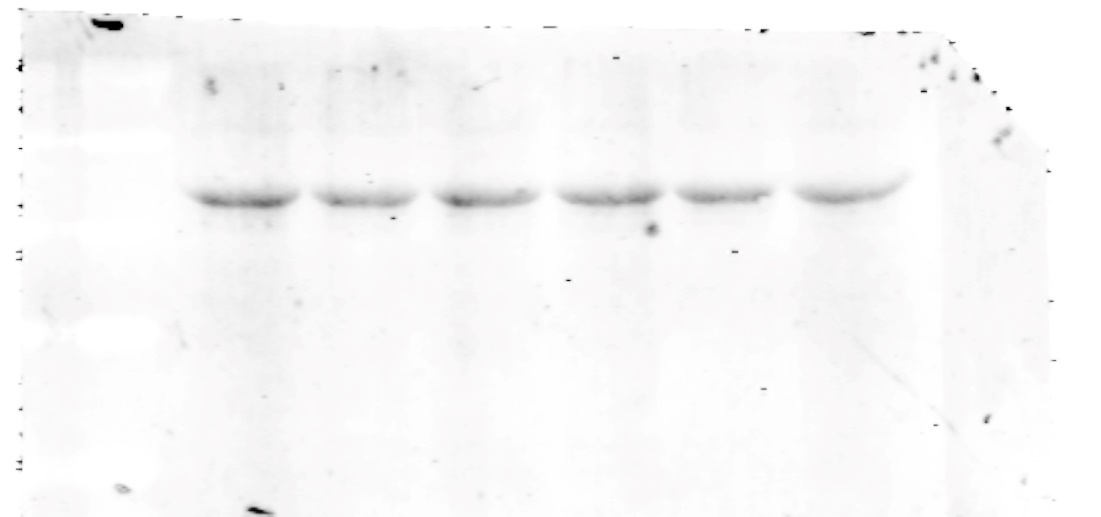


0 5 15 30 45 60（min）

Phos-mpk6

**Figure S5 (c)**: MPK6 blotting of Control treatment for 0-60 min.


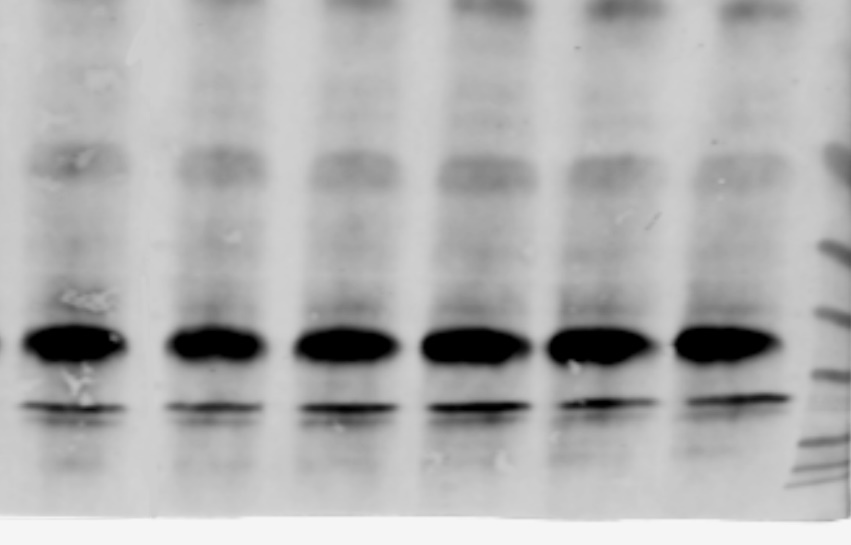


MPK6

0 5 15 30 45 60（min）

**Figure S5 (d)**: MPK6 blotting of C10-HSL treatment for 0-60 min.
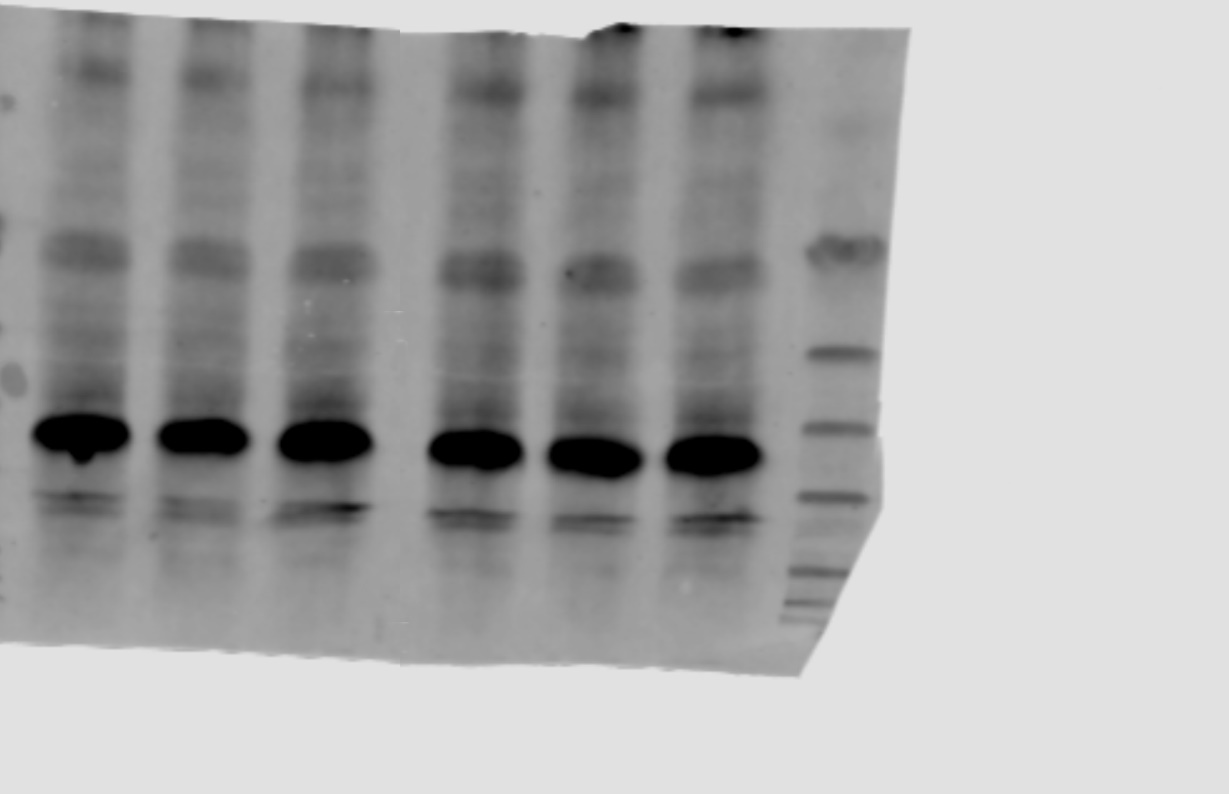


0 5 15 30 45 60（min）

MPK6

**Figure S6 (a)**: MPK6 blotting of C10-HSL, C10-HSL plus CAT, Control and CAT


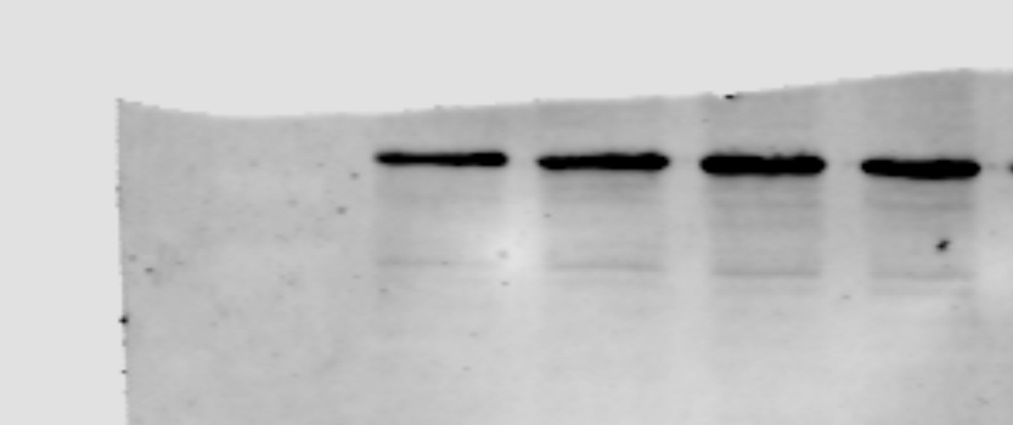


MPK6

C10 C10+CAT Con CAT

**Figure S6 (b)**: Phos-MPK6 blotting of C10-HSL, C10-HSL plus CAT


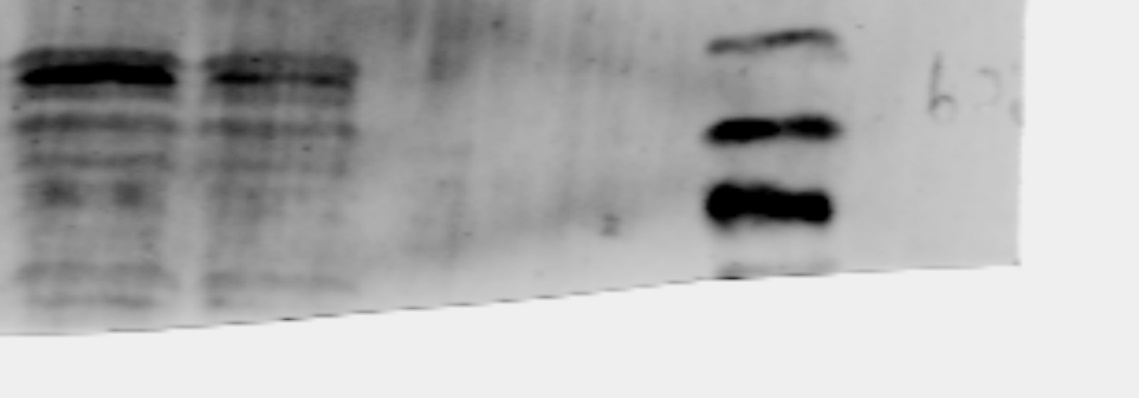


pMPK6

**C10 C10+CAT**

**Figure S6 (c)**: Phos-MPK6 blotting of Con and CAT


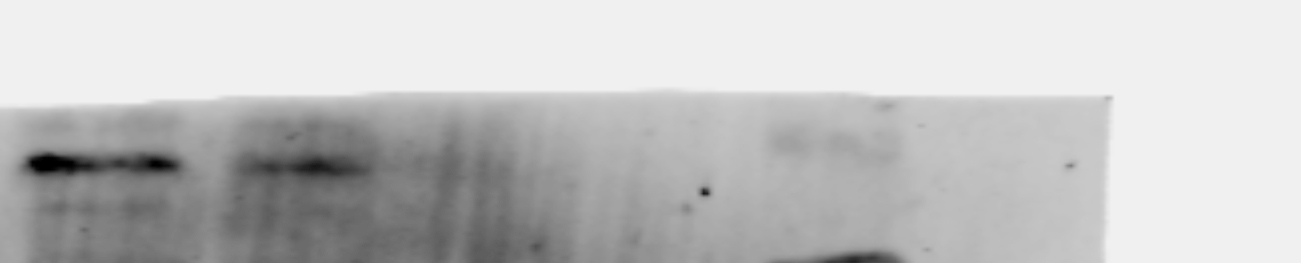


pMPK6

Con CAT
